# Cardio-renal Exosomes in Myocardial Infarction Serum Regulate Proangiogenic Paracrine Signaling in Adipose Mesenchymal Stem Cells

**DOI:** 10.7150/thno.37678

**Published:** 2020-01-01

**Authors:** Lei Gao, Shuya Mei, Shuning Zhang, Qing Qin, Hao Li, Yiteng Liao, Huimin Fan, Zhongmin Liu, Hongming Zhu

**Affiliations:** 1 Research Institute of Heart Failure, Shanghai East Hospital, Tongji University School of Medicine, Shanghai, China; 2 Institute for Regenerative Medicine, Shanghai East Hospital, Tongji University School of Medicine, Shanghai, China; 3 Department of ICU, South Campus, Renji Hospital, School of Medicine, Shanghai Jiao Tong University, Shanghai, China; 4 Department of Cardiology, Zhongshan Hospital, Fudan University, Shanghai Institute of Cardiovascular Disease, Shanghai, China.; 5 Department of Cardiology, Shanghai Tenth People's Hospital, Tongji University School of Medicine, Shanghai, China

**Keywords:** myocardial infarction, mesenchymal stem cell, angiogenesis, microRNA, microparticles

## Abstract

**Rationale:** Mesenchymal stem cells (MSCs) play important roles in tissue repair and regeneration. However, the molecular mechanisms underlying MSCs activation remain largely unknown, thus hindering their clinical translation. Exosomes are small vesicles that act as intercellular messengers, and their potential for stem cell activation in pathological conditions has not been fully characterized yet. Here, we aim to investigate whether serum exosomes are involved in the remote activation of MSCs after myocardial infarction (MI).

**Methods:** We established MI mouse model by ligating the left anterior descending branch of the coronary artery. Afterwards, serum exosomes were isolated from control (Con Exo) and MI mice (MI Exo) by differential centrifugation. Exosomes were characterized through transmission electron microscopy and nanoparticle tracking analysis. The cell proliferation rate was evaluated by CCK-8 and EdU incorporation assays. Exosomal miRNA and protein levels were assessed using qRT-PCR and western blotting, respectively. VEGF levels in the supernatant and serum were quantified by ELISA. Matrigel plug and tube formation assays were used to evaluate angiogenesis. To explore miR-1956 roles, overexpression and knock-down experiments were performed using mimic and inhibitor, respectively. Finally, miR-1956 target genes were confirmed using the luciferase reporter assay.

**Results:** Both types of exosomes exhibited typical characteristics and could be internalized by adipose-derived MSCs (ADMSCs). MI Exo enhanced ADMSCs proliferation through the activation of ERK1/2. Gain- and loss-of-function studies allowed the validation of miR-1956 (enriched in MI Exo) as the functional messenger that stimulates ADMSCs-mediated angiogenesis and paracrine VEGF signaling, by downregulating *Notch-1*. Finally, we found that the ischemic myocardium and kidney may be the main sources that release serum exosomes after MI.

**Conclusions:** Cardio-renal exosomes deliver miR-1956 and activate paracrine proangiogenic VEGF signaling in ADMSCs after MI; this process also involves Notch-1, which functions as the core mediator.

## Introduction

Ischemic injury caused by arterial occlusion is the leading cause of morbidity and mortality worldwide 1. Although advances in surgical techniques have made it possible to restore the blood flow through many great vessels, these techniques generally fail to reestablish the microvascular network that is essential for complete functional recovery 2. Angiogenesis, the process through which new capillaries are formed from pre-existing blood vessels, is crucial for tissue revascularization and repair after ischemia 3. Mesenchymal stem cells (MSCs) are multipotent stromal cells present in many adult tissues such as the bone marrow and adipose tissue. Growing evidence supports the vital contribution of MSCs to angiogenesis 4, 5. Uncovering the underlying mechanisms that activate and direct endogenous MSCs to adopt a proangiogenic phenotype after ischemia is of great interest both for translational applications and mechanistic studies.

Exosomes are endosome-derived small vesicles (30-150 nm) that are released by producer cells into the extracellular environment and subsequently internalized by recipient cells 6. As vehicles for proteins and microRNAs (miRNAs), exosomes play important roles in intercellular communication, both locally and systemically 7, 8. After internalization, the exosomal content is released and plays various roles inside recipient cells, thus regulating cell function and phenotype 9-12. Although serum exosomes from peripheral blood have been investigated for biomarker identification 13, 14, their potential to mediate targeted delivery and activate stem cells in pathological conditions has not been fully characterized yet.

Therefore, we sought to verify whether serum exosomes may transport proangiogenic signals to remote MSCs after ischemia and identify the molecular pathways regulating the phenotype of MSCs in this context. To tackle these questions, we established a murine experimental model of myocardial infarction (MI) and examined the behavior of abdominal adipose-derived MSCs (ADMSCs). Serum exosomes were isolated from control (Con Exo) and MI (MI Exo) mice, and their biological features and regulatory properties affecting MSC-mediated angiogenesis were studied extensively.

## Materials and Methods

### Reagents and antibodies

F12 medium, high-glucose Dulbecco's modified Eagle medium (DMEM), trypsin EDTA, penicillin, streptomycin, MEM nonessential amino acids, GlutaMAX™ supplement, mFGF, and mEGF were purchased from Gibco (Gaithersburg, USA). The Cell-Light EdU DNA cell proliferation kit and riboFECT CP Transfection Kit were obtained from RiboBio (Guangzhou, China). U0126 was purchased from Sigma-Aldrich (St. Louis, MO, USA). Primary antibody against β-actin was purchased from Proteintech (Chicago, USA). Primary antibody against Natriuretic peptides B (BNP, Cat# GB11667) was obtained from Servicebio (Wuhan, China). Primary antibody against Lipocalin 2 (Cat# ARG40936) was purchased from arigo Biolaboratories (Shanghai, China). Phalloidin, DAPI, and primary antibodies against Akt (Cat# 9272), p-Akt (Cat# 4060), MEK1/2 (Cat# 8727), p-MEK1/2 (Cat# 9154), ERK1/2 (Cat# 4695), p-ERK1/2 (Cat# 4370), and HNF4α (Cat# 3113) were purchased from Cell Signaling Technology (Danvers, USA). Primary antibodies against Alix (Cat# ab186429), CD9 (Cat# ab92726), CD63 (Cat# ab217345), Flotillin (Cat# ab133497), CD31 (Cat# ab28364), fatty-acid-binding protein 4 (FABP4, Cat# ab92501), cardiac troponin T (cTnT, Cat# ab8295), CTGF (Cat# ab6992), Nephrin (Cat# ab85379), SP1 (Cat# ab59257), and Notch-1 (Cat# ab52627) were obtained from Abcam (Cambridge, UK). A primary antibody against VEGF (Cat# NB100-664) was purchased from Novus Biologicals (Littleton, USA). Secondary antibodies were obtained from KPL (Washington, USA).

### Animal models

The present study was conducted in accordance with the Guide for the Care and Use of Laboratory Animals published by the National Institutes of Health of the United States (Eighth Edition, 2011). All animal procedures were approved by the Animal Care and Use Committee of Tongji University School of Medicine. Male wild-type C57BL/6 mice (8-10 weeks of age) were purchased from the Shanghai Jiesijie Animal Company (Shanghai, China). To establish murine MI models, mice were anesthetized through sodium pentobarbital intraperitoneal injection (50 mg/kg), then the left anterior descending branch of the coronary artery were ligated as previously described 15. For a standardized moderate infarct size, we ligated the left anterior descending branch at 1.5 mm below the level of the inferior margin of the left auricle. Echocardiography was performed using a 15-Mhz linear-array probe and Sonos 4500 ultrasonograph to evaluate the cardiac function of mice anesthetized using isoflurane inhalation (1.5%-2%). All measurements were performed by the same investigator in a blind manner. All animals were euthanized by CO_2_ inhalation.

### Exosomes isolation

Whole blood samples were obtained from the retro-orbital sinus of control and MI mice. Blood samples were centrifuged (5000 rpm, 10 min), and the serum fraction was collected. As previously introduced 16, serum samples were centrifuged a second time (10,000 g, 10 min) to remove cell debris and aggregates. Afterwards, these samples were ultra-centrifuged at 150,000 g for 2 h in an ultracentrifuge equipped with an MLA-80 rotor (Optima MAX-XP, Beckman Coulter). The supernatant was discarded, while the pellet resuspended in PBS was washed through another ultra-centrifugation step (150,000 g, 2 h). Exosomes isolated from 100 μL of serum were finally resuspended in 50 μL PBS and stored for short-term at -20 °C as previously suggested 17, 18. Con and MI Exo were isolated from control and 3 days MI mice, respectively. For all *in vitro* experiments, the 50 μL exosomes-PBS were accordingly diluted in 1 mL cell culture medium. The number and size of exosomes were assessed by nanoparticle tracking analysis (NTA) using the ZetaView PMX 110 analyzer (Particle Metrix, Meerbusch, Germany) and the corresponding ZetaView 8.04.02 SP2 software. Briefly, resuspended exosomes were diluted in PBS, and NTA measurements were recorded and analyzed at 11 positions. The ZetaView system was calibrated to 110 nm polystyrene particles. The total amount of exosomal proteins was quantified using the BCA protein assay kit (Pierce, USA).

### Transmission electron microscopy

Exosomes were fixed with 2.5% glutaraldehyde overnight at 4 °C. Afterwards, exosomes were loaded onto carbon-coated electron microscopy grids and stained with phosphotungstic acid for 10 min at room temperature. Images were captured using the transmission electron microscope (JEOL, Japan).

### Exosome labelling

Exosomes were incubated with 5 μmol/L calcein-AM at 37 °C for 30 min. For *in vitro* experiments, cells were cultured in medium supplemented with labelled exosomes for 6 h, then fixed with 4% paraformaldehyde (PFA). Cells were then stained with Phalloidin (0.33 μmol/L) and DAPI (5 μg/mL) for 15 min. For *in vivo* experiments, labelled exosomes (100 μL) were injected into the adipose tissue from mouse hind limbs. Exosome internalization was allowed for 12 h, then mice were sacrificed. The adipose tissue was collected, fixed with 4% PFA, ethanol-dehydrated, embedded in paraffin, and sectioned. Tissue sections were stained with FABP4, a specific adipocyte marker, and DAPI and then visualized under a confocal laser microscope (Leica, TCS SP5II STED, USA).

### Cell culture

ADMSCs from the abdominal adipose tissue of C57BL/6 mice were purchased from Cyagen Biosciences (Guangzhou, China). Human embryonic kidney cells (HEK293) and human umbilical vein endothelial cells (HUVEC) were purchased from Shanghai Institute of Biochemistry and Cell Biology. ADMSCs were maintained in F12 medium supplemented with 15% FBS (HyClone, UK), 100 U/mL penicillin, 100 μg/mL streptomycin, MEM nonessential amino acids (1X), GlutaMAX™ supplement (1X), 5 ng/mL mFGF, and 5 ng/mL mEGF. HEK293 and HUVEC were cultured in DMEM supplemented with 10% FBS, 100 U/mL penicillin, and 100 μg/mL streptomycin. Cells were incubated at 37 °C in a humidified atmosphere supplemented with 5% CO_2_.

### Cell proliferation assay

Cells were seeded into 96-well plate and cultured overnight at 37 °C. After the indicated treatments, 10 µL CCK-8 solution (Bimake, China) was added into each well. After an additional incubation at 37 °C for 2 h, the absorbance was measured at 450 nm using the microplate reader (Dynatech, USA).

### EdU assay

The effect of exosomes on cell proliferation was verified using the Cell-Light EdU DNA cell proliferation kit. Briefly, cells were stained with 50 µmol/L of EdU at 37 °C for 2 h. After being fixed with 4% PFA for 30 min, cells were washed with 2 mg/mL glycine and then permeabilized with 0.5% Triton X-100. Cells were reacted with the Apollo reaction cocktail for 30 min, and nuclei were labelled with Hoechst33342. Images were captured using a fluorescence microscope (Leica, DMI3000 B, USA).

### *In vitro* differentiation assay

To explore the effect of Con and MI Exo on the endothelial differentiation of ADMSCs, cells were cultured inside chamber slides in EGM-2 medium (Lonza, USA) to stimulate their endothelial differentiation 19. Con Exo, MI Exo, or equal volumes of PBS were added into the EGM-2 medium, and these induction media was renewed every 3 days. After 10 days of treatment, cells were stained and imaged through confocal microscopy.

### Tube formation assay

The formation of capillary-like structures was determined via using the Matrigel (BD Bioscience, USA). Cells were seeded into the Matrigel-coated 96-well plate, and after required treatments for 5 h, images were captured using a bright-field microscope. The average tube length was quantified using the AngioTool software.

### *In vivo* matrigel plug assay

To test whether MI affected ADMSC-mediated angiogenesis, ADMSCs (5 x 10^6^ cells) were resuspended in 200 μL of ice-cold Matrigel, and implanted subcutaneously on the back of mice, while an equal volume of Matrigel without ADMSCs was implanted as negative control. Subsequently, MI models were established as described above, and the Matrigel plugs were removed for analysis 14 days later.

To evaluate the effects of Con and MI Exo on ADMSC-mediated angiogenesis, ADMSCs (5 x 10^6^ cells) were implanted on the back of mice as described above. Con or MI Exo (20 μg/injection), or equal PBS volumes were injected into the subcutaneous Matrigel plugs on Day 0, Day 5, and Day 10. Finally, the Matrigel plugs were removed for analysis on Day 14.

To determine whether exosomal miR-1956 is responsible for the proangiogenic effect of MI Exo, ADMSCs (5 x 10^6^ cells) were once again implanted on the back of mice. The Matrigel plugs were then injected with MI Exo (20 μg/injection) supplemented with PBS, antagomir-negative control (antagomir-NC, 4 μg/mouse), or antagomir-1956 (4 μg/mouse) on Day 0, Day 5, and Day 10. The Matrigel plugs were removed for analysis after 14 days.

### ELISA assay

VEGF levels in the supernatant serum samples were measured using the mouse VEGF ELISA Kit (Proteintech, USA) according to the manufacturer's instructions. The absorbance was measured at 450 nm using the microplate reader (Dynatech, USA). The concentration of VEGF was determined by interpolation from a standard curve generated in each experiment.

### Histology and immunofluorescence

Matrigel plugs and hearts were collected, fixed with 4% PFA, embedded in paraffin, and sectioned. For immunohistochemical analyses, Matrigel plug sections were stained with primary antibodies against CD31. To identify fibrosis areas, we used the Masson's Trichrome Stain Kit (Solarbio, China) according to the manufacturer's instructions. For immunofluorescence analyses, Matrigel plug and heart sections were stained with anti-VEGF and anti-cTnT antibodies, and DAPI as previously described 20.

### miRNA sequencing and bioinformatics analyses

Con Exo and MI Exo were isolated as described above, and exosomal miRNA-seq analysis was performed by RiboBio (Guangzhou, China) using the Illumina HiSeq 2500 instrument. Bioinformatics analyses including differentially expressed miRNA analysis, prediction of target genes of miRNA, gene ontology (GO) analysis, and Kyoto Encyclopedia of Genes and Genomes (KEGG) pathway enrichment analysis were also performed by RiboBio.

### Luciferase reporter assay

To validate miR-1956 target genes, we performed luciferase reporter assays on HEK293 cells as previously described 21. Briefly, cells were transiently transfected using TransIT-LT1 (Mirus) according to the manufacturer's protocol. A dual-luciferase reporter assay system (Promega) and a Sirius single tube luminometer (Berthold) were used to determine luciferase activity. The *Renilla* luciferase coding vector (Promega) was co-transfected in all cases to evaluate transfection efficiency. Luciferase experiments were performed in quadruplicate and repeated three times independently. Luciferase activity results are presented as relative light units (RLU): the average of *Photinus pyralis* luciferase activity was divided by the average of *Renilla* luciferase activity.

### Cell transfection

The mimic, inhibitor, and corresponding negative control of miR-1956 were synthesized by RiboBio (Guangzhou, China). Briefly, desired mmu-miR-1956 miRNA sequences were synthesized using an Automated Oligonucleotide Solid Synthesizer. Upon completion of the chain assembly, products were obtained after cleavage from the solid phase to solution, deprotection, and lyophilization. The sequences of these products are presented in Table S1. For *in vitro* experiments, cells were transfected with the mimic or inhibitor using the riboFECT CP Transfection Kit according to the manufacturer's instructions.

For *Notch-1* overexpression, control and pCMV-Notch1 (H3176 pLenti-CMV-MCS-HA-3Flag-P2A-EGFPT2APuro) plasmids were purchased from Oobio Corporation (Shanghai, China). Cells were transfected using Lip6000 (Beyotime Institute of Biotechnology, China) following the manufacturer's instructions.

### RNA isolation and quantitative real-time PCR

Total RNA was isolated from ADMSCs using the TRIzol (Invitrogen, USA) extraction method. cDNA libraries were synthesized from extracted mRNA using the Reverse Transcription Reagent kit (Takara, Japan) and random primers, while the cDNA library of miRNA was synthesized using the miRcute Plus miRNA First-Strand cDNA synthesis Kit (TIANGEN, China). Samples were submitted for Real-Time PCR using SYBR Green mix (Takara, Japan), and results were normalized to *Gapdh* (for mRNA) or U6 (for miRNA) expression levels. *Gapdh*, *Cd31*, *Vegf*, and *Notch-1* specific primers were obtained from Sangon Biological Engineering (Shanghai, China), while miR-1956, miR-192-3p, miR-138-5p, and U6 specific primers were obtained from RiboBio. Primer sequences are presented in Table S2. Data were analyzed through the comparative Ct (^ΔΔ^Ct) method to quantify relative gene expression.

### Western blotting

Tissues and cells were lysed in a lysis buffer (Cell Signaling Technology, USA) supplemented with protease inhibitors (Calbiochem, USA) at 4 °C for 30 min, while the exosomes isolated from 100 μL serum were lysed in 20 μL lysis buffer at 4 °C for 10 min. Total protein concentration was quantified using the BCA protein assay kit (Pierce, USA). Western blotting was performed as previously described 22; specific bands were visualized using the LI-COR system (Odyssey, USA), and quantified using the Gel-Pro software (Media Cybernetics, USA).

### Statistical analysis

Results are presented as mean ± standard deviation (SD). Pair groups were compared using the two-tailed Student's t-test. Multiple group comparisons were assessed through One-way ANOVA and Tukey's multiple comparison test. P values < 0.05 were considered significant. All statistical analyses were performed using the GraphPad Prism 7 software (GraphPad Software, Inc., San Diego, USA).

## Results

### Characterization of serum exosomes

**Figure 1:**
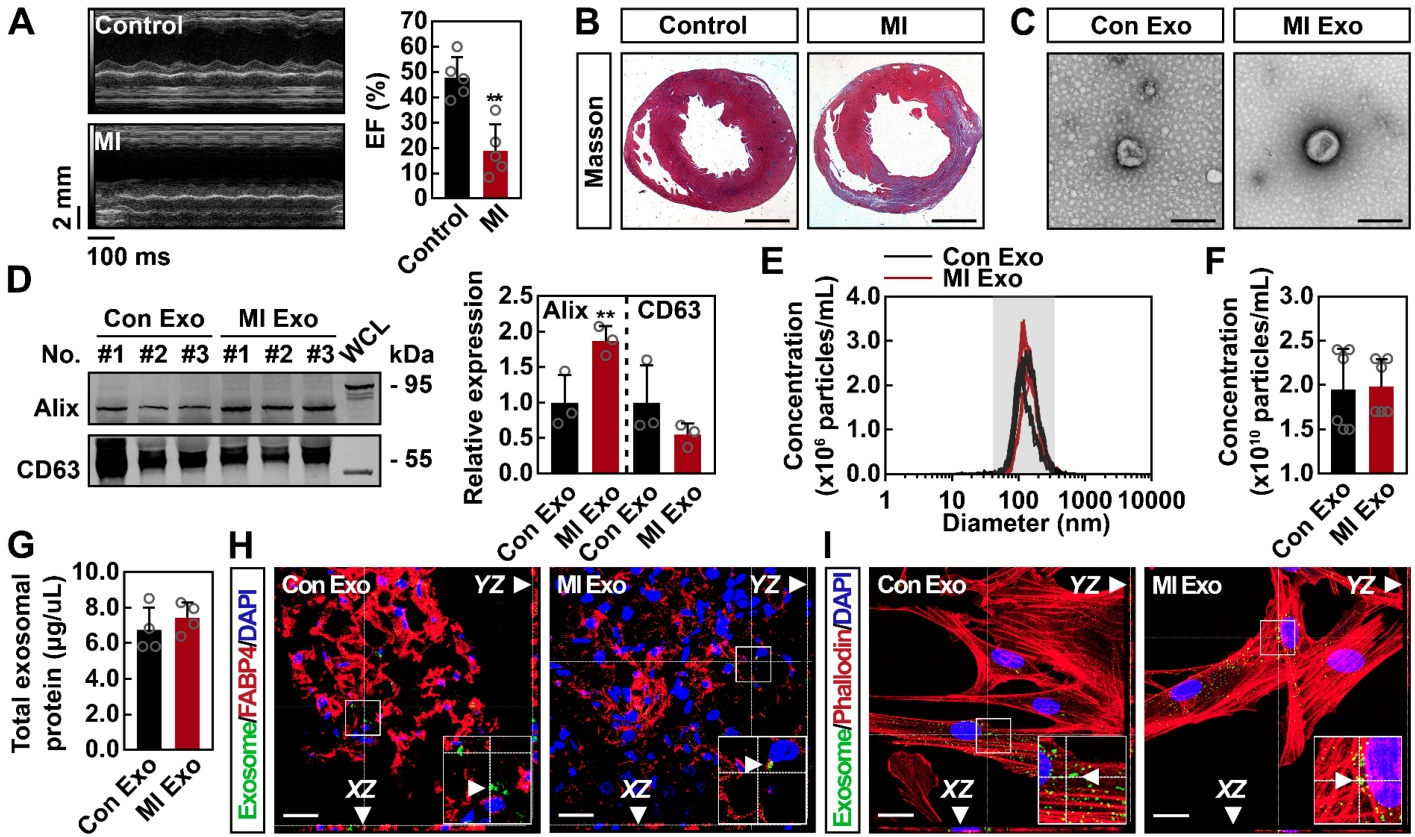
** Characterization and internalization of serum exosomes.** (**A**) Representative images of echocardiography and echocardiographic measurements of EF of control mice and mice with MI for 3 days. MI, myocardial infarction; EF, ejection fraction. n = 5 mice per group. Data are shown as mean ± SD. *, significantly different from control; **, p < 0.01. (**B**) Representative images of Masson's trichrome-stained sections from control mice and mice with MI for 3 days. Scale bar, 1 mm. n = 3 mice per group. (**C**) Transmission electron microscopic analysis of Con Exo and MI Exo. Representative images from two biological replicates are shown. Scale bar, 200 nm. (**D**) Western blot analysis of exosomal marker proteins including Alix and CD63. Exosomal lysates of Con Exo and MI Exo were derived from an equal volume of serum and then loaded. WCL, whole cell lysate derived from ADMSCs. n = 3 mice per group. Data are shown as mean ± SD. *, significantly different from Con Exo; **, p < 0.01. (**E**) Size distribution analysis of Con Exo and MI Exo. Representative image from six biological replicates is shown. (**F**) Quantification of the number of Con Exo and MI Exo isolated from an equal volume of serum. n = 6 mice per group. Data are shown as mean ± SD. (**G**) The concentration of total exosomal protein quantified by BCA. Con Exo and MI Exo were isolated from an equal volume of serum. n = 4 mice per group. Data are shown as mean ± SD. Confocal microscopic analysis of the internalization of Con Exo and MI Exo by adipose tissues (**H**) and ADMSCs (**I**), respectively. Exosomes were labelled with calcein-AM (green), cell skeleton was labelled with phalloidin (red), adipocytes were labelled with FABP4 (red), and nuclei were labelled with DAPI (blue). Representative images from two biological replicates are shown. Scale bar, 20 μm; arrows indicate exosomes. Unpaired Student's t-test was performed (**D, F, and G**).

To determine whether serum exosomes deliver proangiogenic signals after ischemia, MI models were established. Three days after the surgical procedure, MI mice exhibited significantly decreased cardiac function (Figures 1A and S1A) and dramatically increased myocardial fibrosis (Figures 1B and S1B). Serum exosomes were isolated from control (Con Exo) and MI (MI Exo) mice and characterized through electron microscopy and NTA assays. Transmission electron microscopy showed that both types of exosomes were round-shaped vesicles with diameters ranging between 100-150 nm (Figure 1C). The expression of exosomal markers was also studied. As shown in Figure 1D, both Con and MI Exo expressed representative markers such as Alix and CD63; moreover, they also expressed two additional exosomal markers: CD9 and Flotillin (Figure S1C). NTA results revealed that the size distribution of Con and MI Exo ranged between 30-150 nm (Figure 1E). In addition, there was no significant difference between the average diameter of Con Exo (140.35 nm) and MI Exo (150.9 nm) (Figure S1D). BCA and NTA assays were used to quantify the exosomal protein concentration as previously described 23, 24. As shown in Figures 1F-G, we found that the particle density was 1.95 x 10^10^ particles/mL for Con Exo and 1.98 x 10^10^ particles/mL for MI Exo, while the total protein amount was 6.7 μg/μL for Con Exo and 7.4 μg/μL for MI Exo. Therefore, there was no significant difference between the total protein concentrations of these two types of exosomes. Since the number of particles was not significantly different between Con Exo and MI Exo, the increased expression of exosomal Alix could be one of MI Exo features (Figure 1D), as exosomes derived from different cellular sources express various levels of exosomal markers 6, 25, 26. Collectively, the exosomal characteristics observed in the present study were consistent with those previously reported 27-30. To evaluate exosome internalization, calcein-AM labelled exosomes were injected into the adipose tissue, and their internalization was assessed 12 h later. As shown in Figures 1H-I, calcein-AM-labelled exosomes were localized in the abdominal adipose tissue, and further co-culture studies confirmed exosomes internalization by ADMSCs. No remarkable difference was observed between the uptake efficiency of Con and MI Exo in both ADMSCs and the adipose tissue. Taken altogether, these results confirm that both types of serum-isolated exosomes exhibit typical characteristics and can be internalized both *in vitro* and *in vivo*.

### MI Exo enhance the proliferation rate of ADMSCs *in vitro*

**Figure 2:**
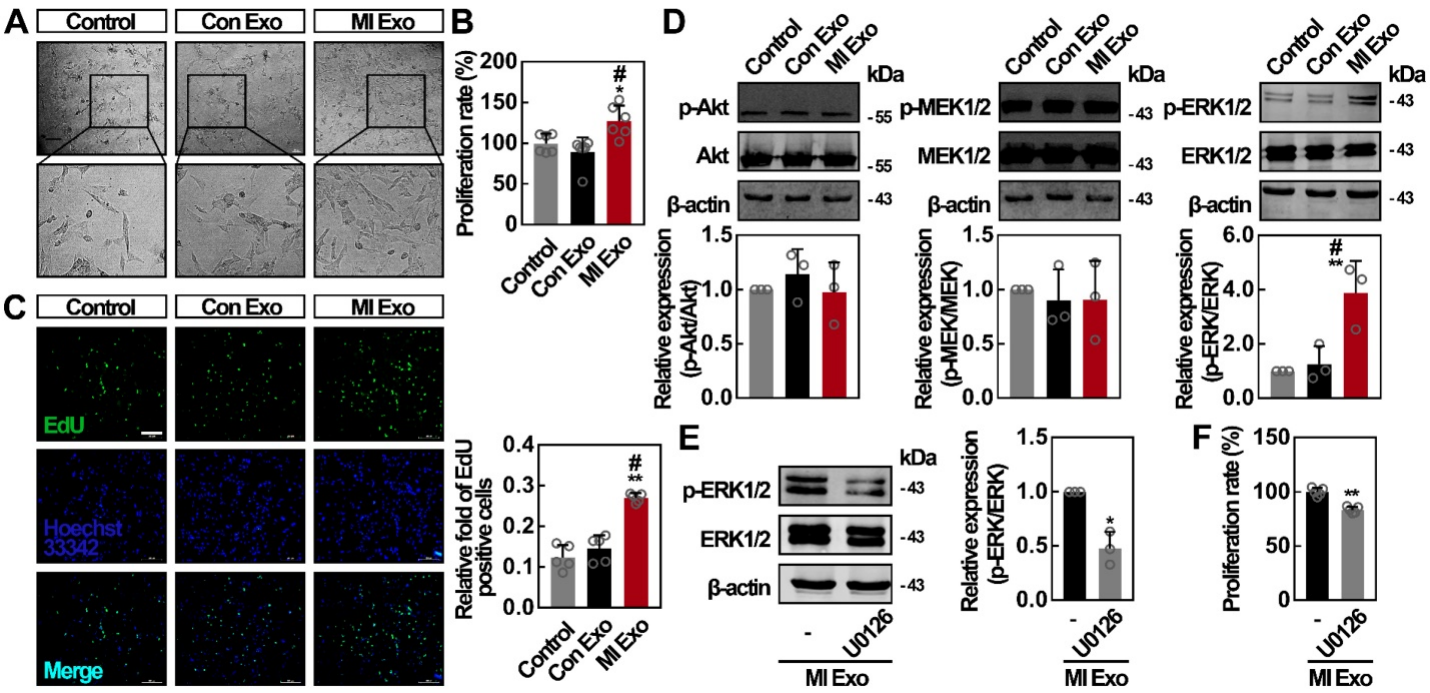
** MI Exo promotes ADMSCs proliferation *in vitro* partly via ERK1/2 activation.** (**A**) Inverted contrast phase microscopy showed that the number of ADMSCs was increased after treatment with MI Exo for 24 h compared with the results for control and Con Exo. Scale bar, 80 μm. (**B**) The proliferation rate of ADMSCs was measured using CCK-8 assay after treatment with Con Exo and MI Exo for 24 h. Data are shown as mean ± SD. *, significantly different from control; *, p < 0.05. #, significantly different from Con Exo; #, p < 0.05. Three independent experiments were performed. (**C**) EdU incorporation analysis of ADMSCs after treatment with Con Exo and MI Exo for 24 h. The EdU incorporation rate was expressed as the ratio of EdU-positive cells to total Hoechst33342-positive cells. Scale bar, 200 μm. Data are shown as mean ± SD. *, significantly different from control; **, p < 0.01. #, significantly different from Con Exo; #, p < 0.05. Two independent experiments were performed. (**D**) Western blot analysis of p-Akt and Akt; p-MEK1/2 and MEK1/2; p-ERK1/2 and ERK1/2 after treatment with Con Exo and MI Exo for 24 h. Representative images of three independent experiments are shown. p-, phosphorylated. Data are shown as mean ± SD. *, significantly different from control; **, p < 0.01. #, significantly different from Con Exo; #, p < 0.05. After being treated with MI Exo and 10 μmol/L U0126 for 24 h, the expression of p-ERK1/2 and ERK1/2 of ADMSCs was determined using western blotting (**E**), and the proliferation rate was measured using CCK-8 assay (**F**). For **E**, representative images of three independent experiments are shown. For **F**, two independent experiments were performed. Data are shown as mean ± SD. *, significantly different from MI Exo; *, p < 0.05; **, p < 0.01. One-way ANOVA followed by Tukey's post-test was performed (**B**-**D**). Unpaired Student's t-test was performed (**E** and **F**).

To evaluate the activity of serum exosomes on ADMSCs, we first studied cell proliferation rates after Con or MI Exo treatment for 24 h. For *in vitro* experiments, we used the following exosome concentrations: 9.75 x 10^8^ particles/mL for Con Exo, and 9.8 x 10^8^ particles/mL for MI Exo. We found that cell numbers were significantly increased after MI Exo treatment. In addition, MI Exo significantly increased ADMSC viability compared to the PBS control and Con Exo treatment (Figures 2A-B). Similar trends were observed through EdU incorporation assays. As shown in Figure 2C, MI Exo-treated ADMSCs showed the largest number of EdU-positive cells compared to other treatments. It has been proven that Akt and MEK1/2/ERK1/2 signaling are two critical parallel pathways regulating cell proliferation 31. We then studied whether Akt and MEK1/2/ERK1/2 signaling pathways were activated after MI Exo treatment. As shown in Figure 2D, neither MI Exo, nor Con Exo treatment significantly altered p-Akt/Akt and p-MEK1/2/MEK1/2 ratios. Interestingly, we found that MI Exo significantly increased ERK1/2 phosphorylation level, resulting in a tripled p-ERK1/2/ERK1/2 ratio compared with the one observed after Con Exo treatment. Treatment with U0126, an ERK1/2 inhibitor 32, suppressed MI Exo mediated activation of ERK1/2 (Figure 2E), and attenuated cell proliferation after MI Exo treatment (p < 0.05, Figure 2F). Collectively, these results demonstrate that MI Exo enhance the proliferation rate of ADMSCs through the activation of ERK1/2.

### MI Exo stimulate VEGF paracrine signaling in ADMSCs

**Figure 3:**
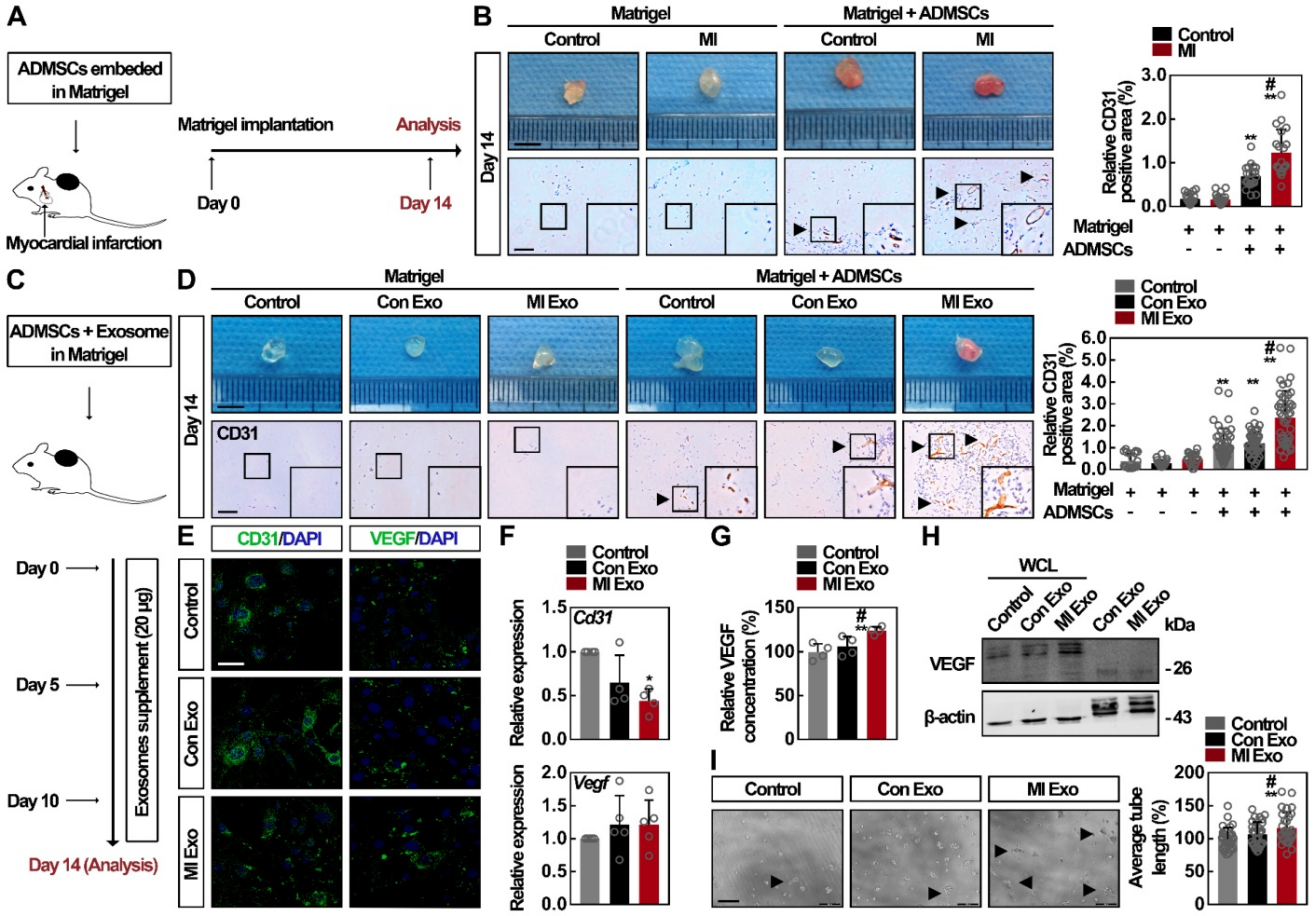
** MI Exo enhance the ADMSC-mediated angiogenesis by promoting VEGF paracrine but not endothelial differentiation.** (**A**) ADMSCs embedded in Matrigel were implanted subcutaneously on the back of mice, and MI models were then established. The implantation of cell-free Matrigel was served as negative control, and Matrigel plugs were removed on Day 14. (**B**) Representative macroscopic images and CD31 immunohistochemical staining of Matrigel plugs removed on Day 14. Scale bar, 5 mm (for macroscopic images); Scale bar, 100 μm (for immunohistochemical images); arrows indicate blood vessels. n = 4 mice per group. CD31 quantification is shown. Data are shown as mean ± SD. *, significantly different from the control group bearing with cell-free Matrigel; **, p < 0.01. #, significantly different from control group bearing with ADMSCs-embedded Matrigel; #, p < 0.05. (**C**) ADMSCs embedded in Matrigel were implanted subcutaneously on the back of mice. Con Exo and MI Exo (20 μg/each injection) were injected into the subcutaneous Matrigel plugs on Day 0, Day 5, and Day 10, respectively. PBS injection was served as the control. These Matrigel plugs were removed on Day 14. (**D**) Representative macroscopic images and CD31 immunohistochemical staining of Matrigel plugs removed on Day 14. Scale bar, 5 mm (for macroscopic images); Scale bar, 100 μm (for immunohistochemical images); arrows indicate blood vessels. n = 4 mice per group for the groups with cell-free Matrigel; n = 5 mice per group for the groups with ADMSCs-embedded Matrigel; CD31 quantification is shown. Data are shown as mean ± SD. *, significantly different from the control group bearing with cell-free Matrigel; **, p < 0.01. #, significantly different from control group bearing with ADMSCs-embedded Matrigel; #, p < 0.05. After being treated with Con Exo or MI Exo for 10 days following endothelial induction by EGM-2 medium, the protein and relative mRNA level for *Cd31* and *Vegf* was assessed by confocal microscopy (**E**) and qRT-PCR (**F**), respectively. For **E**, cells were labelled with CD31 and VEGF (green), and nuclei were stained with DAPI (blue). Scale bar, 50 μm. Representative images of two independent experiments are shown. For **F**, data are shown as mean ± SD of four independent experiments for *Cd31* and five independent experiments for *Vegf*. After being treated with Con Exo or MI Exo for 3 days, the level of secreted VEGF was detected by ELISA (**G**), and the expression of cellular VEGF was determined by western blotting (**H**). For **G**, two independent experiments were performed. Data are shown as mean ± SD. *, significantly different from control; **, p < 0.01. #, significantly different from Con Exo; #, p < 0.05. For **H**, representative images of three independent experiments are shown. Exosomal lysates of Con Exo and MI Exo were derived from an equal volume of serum. WCL, whole cell lysate derived from ADMSCs. (**I**) Tube formation analysis of ADMSCs after treatment with Con Exo and MI Exo for 5 h. Average tube length was quantified. Data are shown as mean ± SD from three independent experiments. *, significantly different from control; **, p < 0.01. #, significantly different from Con Exo; #, p < 0.05. One-way ANOVA followed by Tukey's post-test was performed (**B**, **D**, **F**, **G** and **I**).

To test whether circulating exosomes regulate ADMSC-mediated angiogenesis *in vivo* after MI, which is a critical process for tissue repair after ischemia 33, 34, we packaged ADMSCs into Matrigel (ADMSC-gel) and implanted them subcutaneously on the back of mice. Two kinds of animal models were adopted: MI (Figure 3A) and mice with MI Exo supplement (Figure 3C). At Day 14, MI mice showed significantly decreased cardiac function and increased myocardial fibrosis, whereas no significant cardiac alteration could be attributed to the transplantation of ADMSCs (Figure S2A). Consequently, CD31 immunohistochemical analysis showed that more vessel-like structures had formed in the ADMSC-gel in MI mice (Figure 3B) 35. Similarly, MI Exo treatment induced the formation blood vessels in the ADMSC-gel, while no vessel-like structures could be observed in the Matrigel-only implanted control mice (Figure 3D). Altogether, these results indicate that ADMSC-mediated angiogenesis is stimulated after MI, and serum exosomes (MI Exo) mediate this activation. The major contribution of ADMSCs in angiogenesis is due to their endothelial differentiation and the proangiogenic paracrine signaling 33, 36; therefore, further studies were designed to explore the underlying mechanism involved in this context. Growth factor-enriched EGM-2 medium was used to induce endothelial differentiation 19, and CD31 and VEGF expression levels were evaluated. As shown in Figures 3E-F, after the 10-days of endothelial induction, we found that neither one of the two types of exosomes could significantly alter the expression levels of CD31 and VEGF in the differentiation medium. Since these two types of exosomes failed to promote ADMSC differentiation, we then studied their activity on proangiogenic paracrine signaling. We found that the levels of secreted VEGF (Figure 3G) and cellular VEGF (Figure 3H) were significantly increased after MI Exo treatment for 3 days. Moreover, there was no significant difference between the amount of exosomal VEGF from Con and MI Exo, suggesting that MI Exo enhance VEGF expression in ADMSCs though the delivery of other molecules than VEGF. Tube formation assays were used to evaluate the effects of Con and MI Exo on ADMSC-mediated angiogenesis *in vitro*. As shown in Figure 3I, the formation of capillary-like structures was significantly increased after MI Exo treatment, compared with Con Exo condition. Collectively, these results suggest that MI Exo enhance ADMSC-mediated angiogenesis by stimulating paracrine VEGF signaling.

### MI Exo transfer miR-1956 to regulate VEGF signaling in ADMSCs

**Figure 4:**
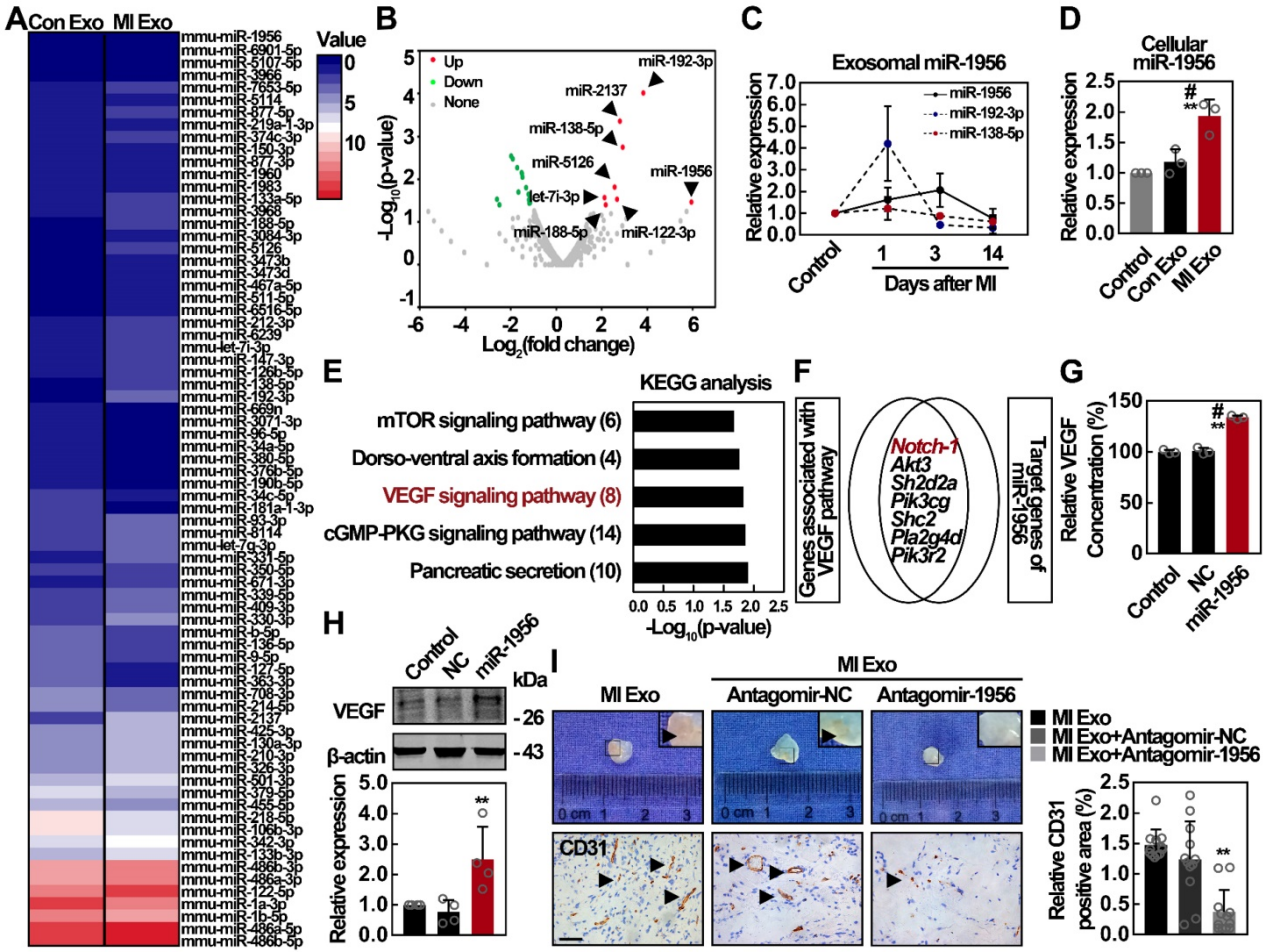
** The proangiogenic effect of MI Exo is largely mediated by exosomal miR-1956.** (**A**) Supervised clustering of miRNAs identified in Con Exo and MI Exo. (**B**) Volcano plot of differentially expressed miRNAs between Con Exo and MI Exo. Red, significantly upregulated miRNAs in MI Exo; green, significantly downregulated miRNAs in MI Exo; gray, no significant difference. Fold change > 2 and p < 0.05 were considered significant. The sequencing samples of Con Exo and MI Exo were isolated from six mice. (**C**) Relative expression of miR-1956, miR-192-3p, and miR-138-5p in Con Exo and MI Exo as assessed by qRT-PCR. Data are shown as mean ± SD of at least three independent experiments. (**D**) Relative expression of miR-1956 in ADMSCs after treatment with Con Exo and MI Exo for 24 h as assessed by qRT-PCR. Data are shown as mean ± SD of three independent experiments. *, significantly different from control; **, p < 0.01. #, significantly different from Con Exo; #, p < 0.05. (**E**) KEGG pathway analysis of the target genes of 8 significantly upregulated miRNAs in MI Exo. Top 5 enriched pathways are indicated. (**F**) Venn diagram comparison of the genes associated with VEGF pathway and the target genes of miR-1956. After transfection with 50 nmol/L miR-1956 mimic for 3 days, the levels of secreted and cellular VEGF were evaluated using ELISA (**G**) and western blotting (**H**), respectively. For **G**, the experiment was performed in triplicate. Data are shown as mean ± SD. *, significantly different from control; **, p < 0.01. Fog **H**, representative images of four independent experiments are shown. Data are shown as mean ± SD. *, significantly different from control; **, p < 0.01. ADMSCs embedded in Matrigel were implanted subcutaneously on the back of mice. Mice were consecutively injected with MI Exo (20 μg/each injection) accompanied by PBS, antagomir-negative control (antagomir-NC, 4 μg per mice) or antagmir-1956 (4 μg per mice) on Day 0, Day 5, and Day 10, respectively. The Matrigel plugs were removed on Day 14. (**I**) Representative macroscopic images and CD31 immunohistochemical staining of Matrigel plugs. Scale bar, 100 μm; arrows indicate blood vessels. n = 3 mice per group. CD31 quantification is shown. Data are shown as mean ± SD. *, significantly different from MI Exo; **, p < 0.01. One-way ANOVA followed by Tukey's post-test was performed (**D**, **G**, **H**, and **I**).

Exosomes play a vital role in intercellular communication, and their functions depend mainly on their internal contents 28. Therefore, we analyzed miRNA contents of both Con and MI Exo through miRNA sequencing (miR-seq), and the supervised clustering of the identified miRNAs was analyzed (Figure 4A). Using 2-fold change and p < 0.05 as the threshold cutoff, we found 24 miRNAs with significantly different distribution between Con and MI Exo, including 8 upregulated and 16 downregulated miRNAs (Figure 4B). The expression of the top 3 significantly upregulated miRNAs from MI Exo, including miR-1956, miR-192-3p, and miR-138-5p, was quantified by qRT-PCR. As shown in Figure 4C, there was a steady increase in the expression of miR-1956 during the first 3 days after MI, followed by a dramatic decrease at Day 14. Alternatively, miR-192-3p expression was dramatically increased at Day 1, and then progressively decreased. We also found that cellular miR-1956 was significantly upregulated after MI Exo treatment for 3 days (Figure 4D). Afterwards, we performed target gene prediction for the upregulated miRNAs, Gene Ontology (GO - Figure S3) and Kyoto Encyclopedia of Genes and Genomes (KEGG) analyses (Figure 4E). Interestingly, 7 out of the 8 predicted targets of miR-1956 were involved in the VEGF signaling pathway (Figure 4F). We thus hypothesized that MI Exo-enriched miR-1956 might regulate VEGF signaling in ADMSCs. Accordingly, miR-1956 overexpression by mimic transfection significantly increased the expression and secretion of VEGF (Figures 4G-H), without impacting the ADMSC proliferation rate or phosphorylation of ERK1/2 (Figure S4). Antagomir targeting miR-1956 (antagomir-1956) was used to evaluate the proangiogenic effect of exosomal miR-1956 in Matrigel plug assays 37. As shown in Figure 4I, antagomir-1956 significantly attenuated the proangiogenic effect of MI Exo, as revealed by the quantification of CD31-positive areas. Altogether, these results demonstrate that MI Exo-enriched miR-1956 regulates VEGF signaling in ADMSCs.

### miR-1956 regulates VEGF through Notch-1

**Figure 5:**
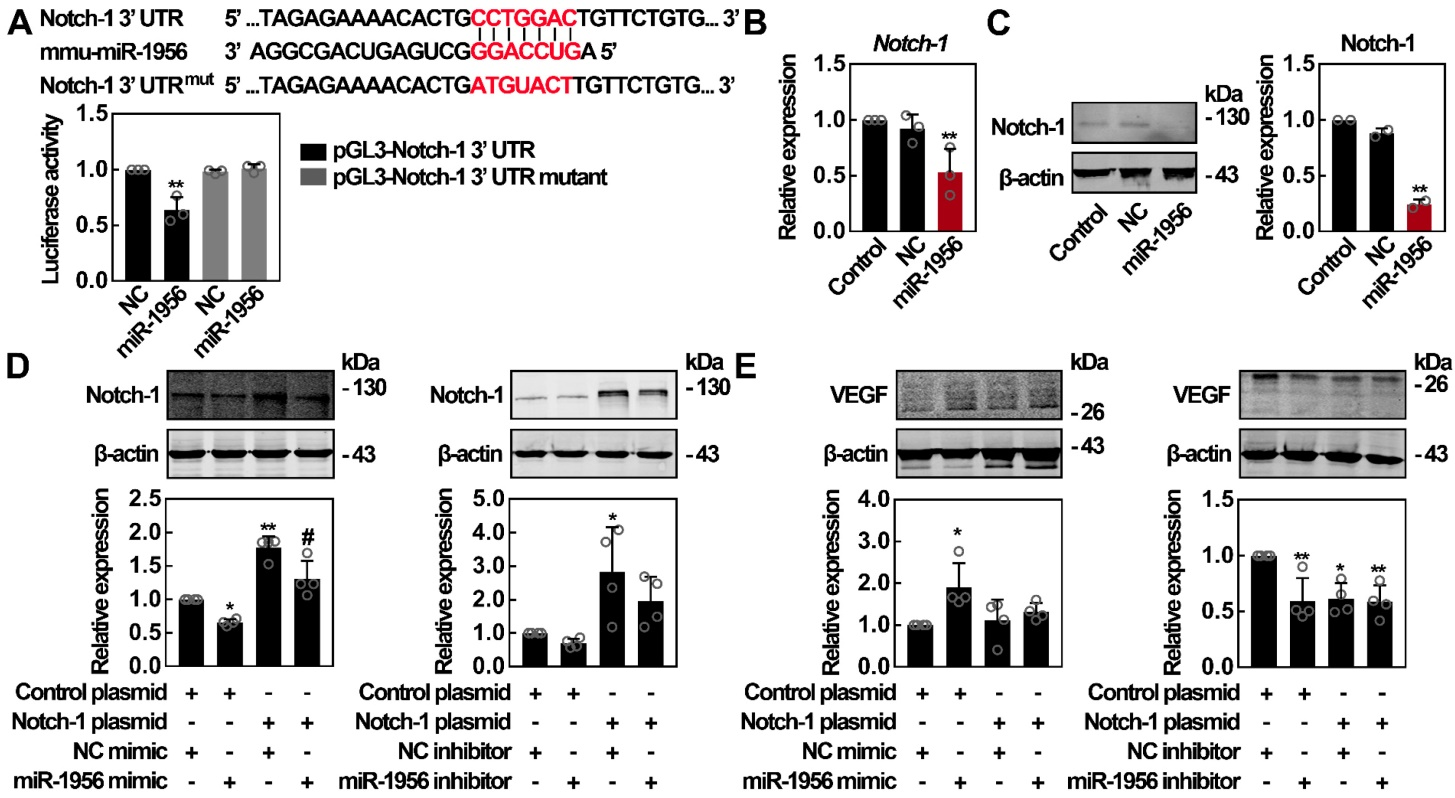
** Notch-1 is identified as an intermediate molecule in the miR-1956-VEGF axis.** (**A**) *Notch-1* is a target gene for miR-1956. The sequence of miR-1956, the potential binding site at the 3' UTR of *Notch-1* mRNA, and the nucleotides mutated in the Notch-1-3' UTR mutant are shown. HEK293 cells were transiently cotransfected with miR-1956 mimic or NC using luciferase reporter vectors. The luciferase activity was normalized to the activity of Renilla luciferase. Data are shown as mean ± SD from three independent experiments. *, significantly different from NC; **, p < 0.01. After transfection with 50 nmol/L of miR-1956 mimic for 3 days, the mRNA level of *Notch-1* was determined using qRT-PCR (**B**), and the protein expression of Notch-1 was measured using western blotting (**C**). For **B**, data are shown as mean ± SD from three independent experiments. *, significantly different from control; **, p < 0.01. For **C**, representative images of two independent experiments are shown. Data are shown as mean ± SD. *, significantly different from control; **, p < 0.01. ADMSCs were transfected with 2.5 μg Notch-1 plasmid and 50 nmol/L miR-1956 mimic or 100 nmol/L miR-1956 inhibitor for 3 days, the intracellular expression of Notch-1 (**D**) and VEGF (**E**) was detected by western blotting. For **D** and **E**, representative images of four independent experiments are shown. Data are shown as mean ± SD. *, significantly different from the control group transfected with control plasmid and NC mimic or inhibitor; *, p < 0.01; **, p < 0.01. #, significantly different from the group transfected with Notch-1 plasmid and NC mimic or inhibitor; #, p < 0.05. One-way ANOVA followed by Tukey's post-test was performed (**A**-**E**).

It has been reported that Notch-1 inhibition can upregulate VEGF expression 38; interestingly, *Notch-1* is a predicted target of miR-1956 (Figure 4D). Therefore, we constructed luciferase vectors containing the wild-type or mutant 3'-UTR sequence of *Notch-1*. As shown in Figure 5A, miR-1956 mimic significantly decreased the relative luciferase activity of the wild-type vectors, while the luciferase activity of mutant vectors was not altered by miR-1956. These findings suggest that miR-1956 specifically binds to the 3'-UTR of *Notch-1* mRNA. Using qRT-PCR and western blotting, we found that miR-1956 overexpression significantly reduced both *Notch-1* mRNA and protein levels (Figures 5B-C). Considering the specific regulatory activity of miR-1956 on Notch-1 expression, and the close relationship between Notch-1 and VEGF (Figure 4), we thus speculated the existence of a miR-1956-Notch-1-VEGF axis to regulate the behavior of ADMSCs after MI. As shown in Figure 5D, although no significant alteration was observed in presence of miR-1956 antagonist, the expression of *Notch-1* was significantly downregulated by miR-1956 mimic, thus demonstrating that Notch-1 is one of the target genes of miR-1956. For rescue studies, we transfected and overexpressed *Notch-1*; in this case neither miR-1956 mimic, nor the inhibitor could influence VEGF expression in ADMSCs (Figure 5E). Taken altogether, these results demonstrate that VEGF expression is upregulated by miR-1956, and Notch-1, one of miR-1956 targets, may function as a mediator in the miR-1956-VEGF axis.

### Systemic analysis of the exosomal marker expression in various organs after MI

**Figure 6:**
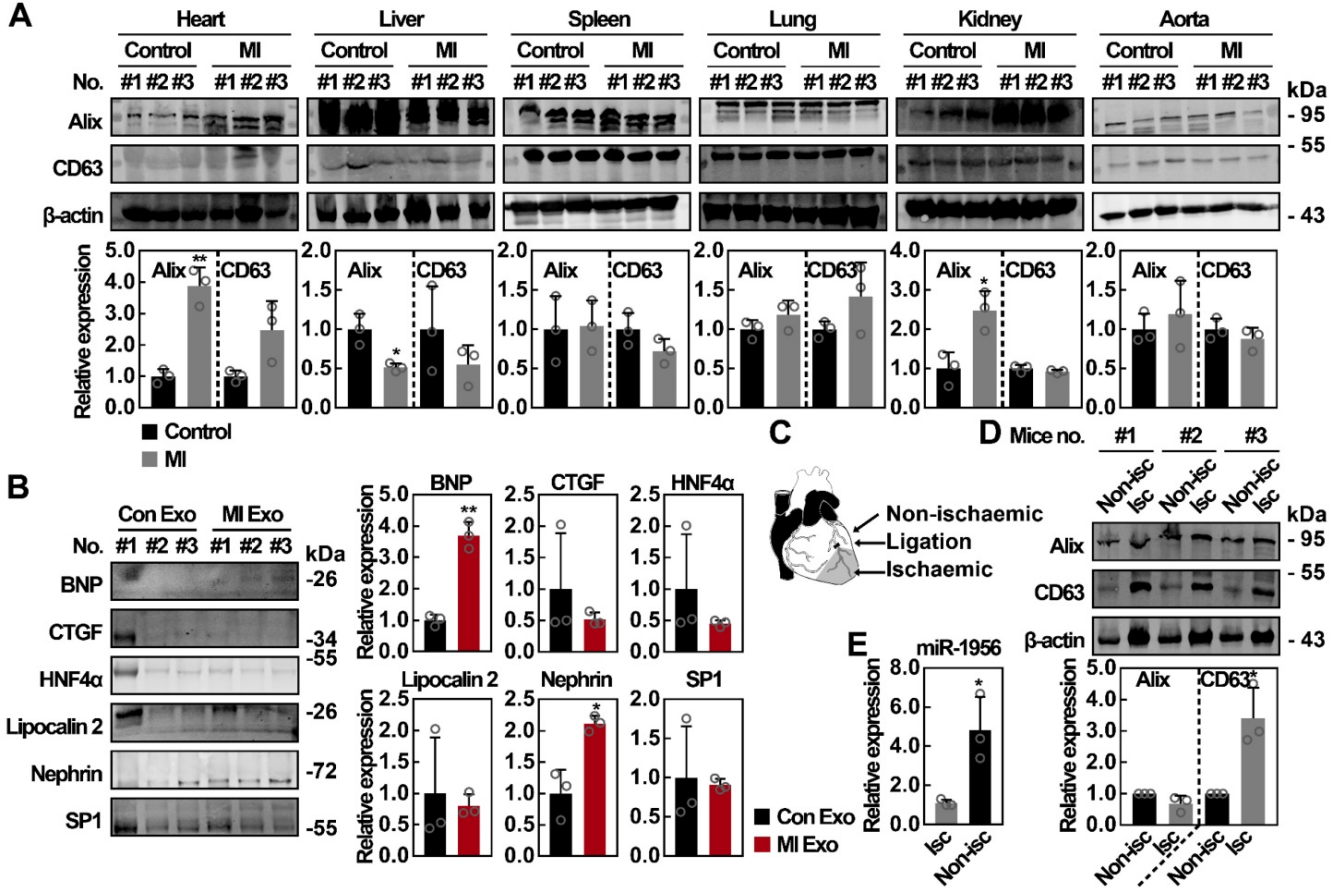
** The organ patterns of exosomes secretion after MI.** (**A**) Western blot analysis of tissue lysates from heart, liver, spleen, lung, kidney, and aorta of control mice and MI mice. n = 3 mice per group. Data are shown as mean ± SD. *, significantly different from control; *, p < 0.05; **, p < 0.01. (**B**) Western blot analysis of organ-specific and nonspecific proteins including BNP, CTGF, HNF4α, Lipocalin 2, Nephrin, and SP1. Exosomal lysates of Con Exo and MI Exo were derived from an equal volume of serum and then loaded. n = 3 mice per group. Data are shown as mean ± SD. *, significantly different from Con Exo; *, p < 0.05; **, p < 0.01. (**C**) Schematic diagram of cardiac regions. (**D**) Western blot analysis of nonischemic and ischemic tissues from MI mice. n = 3 mice per group. Data are shown as mean ± SD. *, significantly different from nonischemic; *, p < 0.05. (**E**) Relative expression of miR-1956 in mice heart tissues including nonischemic and ischemic areas as assessed by qRT-PCR. Data are shown as mean ± SD of three independent experiments. *, significantly different from the infarct area; *, p < 0.05. Unpaired Student's t-test was performed (**A, B, D,** and **E**).

To identify the potential cellular source of functional serum exosomes after MI, we studied the expression of exosomal markers in the heart, liver, spleen, lung, kidney, and aorta following previously described procedures 39, 40. The upregulation of exosomal markers in a tissue indicates that those cells have activated the exosome producing metabolism, therefore becoming potential sources of serum exosomes. As shown in Figure 6A, no significant difference was observed concerning the expression of exosomal markers in spleen, lung, and aorta lysates after MI, while the expression of Alix and CD63 was decreased in liver lysates after MI. However, we found that the expression of Alix in heart and kidney lysates was significantly upregulated, with at least a 2-fold increase after MI (p < 0.05). Previous studies have shown that exosomes carry specific proteins from their cellular source 41-44, therefore the exploration of tissue-specific proteins in circulating exosomes is another method to identify their source. As shown in Figure 6B, the levels of BNP (cardiac marker) 45 and nephrin (renal marker) 46, 47 were significantly increased in MI Exo. However, no significant difference was observed concerning the expression of other markers, such as HNF4α (hepatic marker that can be detected in exosomes) 48, 49, CTGF 50, Lipocalin 2 51, and SP1 52. Furthermore, we investigated the cardiac source of circulating exosomes both in ischemic and non-ischemic areas (Figure 6C) and found that the expression of Alix did not differ between non-ischemic and ischemic areas, while the expression of CD63 was significantly increased in ischemic areas (Figure 6D). Finally, we also evaluated miR-1956 levels in mouse heart tissues. As shown in Figure 6E, compared with ischemic areas, there was a significantly higher level of miR-1956 in the non-ischemic areas. These results indicate that functional serum exosomes released after MI mainly originate from the ischemic myocardium and kidney.

## Discussion

In the present study, we demonstrate that serum exosomes promote paracrine VEGF signaling in ADMSCs through the exosomal miR-1956-Notch-1-VEGF axis. Moreover, we also reveal that the ischemic myocardium and kidney may be the main sources for circulating exosomes after MI, therefore suggesting that serum exosomes serve as an important messenger for the functional regulation of endogenous stem cells.

Angiogenesis, a critical process for the post-ischemic tissue repair, involves various cell types, such as endothelial progenitors and inflammatory cells 53. MSCs have already been proven to contribute to post-ischemic angiogenesis through their differentiation and paracrine signaling activity 5, 54. Many diverse molecules, such as VEGF 55, have been identified as essential factors for the activation and recruitment of MSCs to mediate tissue repair 56. In the present study, we demonstrate that serum exosomes are a novel actor involved in the regulation and activation of stem cells. MI decorates the exosomal content with proangiogenic signals that activate endogenous stem cells. To explore the potential roles of ADMSCs in exosome-enhanced angiogenesis, both their endothelial differentiation and paracrine signaling activity were investigated. As a result, paracrine VEGF signaling, rather than the endothelial differentiation, was shown to contribute to ADMSC-mediated angiogenesis. However, we did not find any significant alteration in the expression of CD31. Since CD31 is a vital surface biomarker of mature endothelial cells 57, our results suggest that MI Exo alone may be insufficient to promote the endothelial differentiation of ADMSCs. We then studied the release of proangiogenic cytokines from ADMSCs 3, 58. Interestingly, MI Exo significantly upregulated *de novo* VEGF expression in ADMSCs, instead of delivering exosomal VEGF (Figures 3G-H).We then observed that VEGF expression in the ischemic heart tissues was markedly increased (Figure S2B), while serum VEGF levels were not significantly different between these groups (Figure S2C). Consistent with these results (Figure 3G-H), the expression of VEGF in Matrigel plugs populated with ADMSCs was significantly increased after MI Exo treatment (Figure S2D). These findings further highlight the important role of VEGF upregulation by circulating exosomes for ADMSC-mediated angiogenesis.

Enhancing circulatory paracrine signals instead of local endothelial differentiation may be of greater benefit after MI. We found that serum exosomes from control mice did not stimulate the proangiogenic phenotype of ADMSCs, therefore MI-modified exosomes may contain key regulators required for this process. Due to the low exosomal protein amount, the exosome-induced phenotypic changes in recipient cells may arise due to the activity of exosomal miRNAs 10. Using deep miRNA-seq analysis, 24 miRNAs differently expressed between Con and MI Exo were identified. miR-1956, miR-192-3p, and miR-138-5p were the top 3 upregulated miRNAs after MI. Among them, the expression of miR-192-3p and miR-138-5p fluctuated in the acute MI. Although we didn't explore their functions here, previous studies reported that miR-192-3p has been identified as an important regulator during hepatitis B virus infection 59, and miR-138-5p mutations have been shown to drive chemotherapeutic drug resistance 60, 61. Notch signaling is a conserved and multifunctional regulator of various cell type- and state-dependent responses 62-64. Activation of Notch signaling results in the proteolytic cleavage and dissociation of Notch receptor's intracellular domain, which is then translocated into the nucleus to regulate target gene expression. Previous studies found that the outcomes of Notch-1 regulation of VEGF vary depending on the cell type 65. For instance, treatment with DAPT, a γ secretase inhibitor suppressing Notch-1 activity, leads to opposite effects on VEGF expression in astrocytes 66 and retinas 38. Recently, a pioneering study has uncovered the feasibility of miRNA-mediated Notch-1 inhibition to upregulate VEGF during the differentiation of induced pluripotent stem cells 67. In the present study, we found that miR-1956 targets Notch-1 to regulate VEGF signaling. Notch-1 overexpression did not impact VEGF expression; however, it successfully rescued miR-1956-induced VEGF upregulation (Figure 5E). It is well known that each miRNA has hundreds mRNAs targets 68, 69; nevertheless, the overexpression of a single target gene has fully rescued the effect of miR-1956 on VEGF expression. Collectively, these findings suggest that Notch-1 is a strong mediator in this signaling axis. Although interesting to explore, the detailed mechanisms underlying how Notch-1 regulates VEGF remain largely unclear.

Circulating exosomes have been recognized as important regulators and potential biomarkers for various diseases 70-73, yet their uncertain cellular origin has been a barrier for their clinical translation. Theoretically, all cells that are in contact with the circulatory system can be a source of circulating exosomes. Previous studies have demonstrated that exosomes carry specific proteins from their cellular source 41-44; thus, identifying tissue-specific proteins in circulating exosomes is another approach to locate their origin. Herein, we demonstrate that the functional circulating exosomes may be released from the heart and kidney after MI. We obtained complementary results through the analysis of exosome metabolism genes (Alix and CD63) and cellular specific markers (BNP and nephrin). In addition, considering the active exosomes secretion (Figure 6D) and lower miR-1956 levels (Figure 6E) observed in the ischemic myocardium, an interesting hypothesis would be that the ischemic areas exhibit lower miR-1956 levels because their miR-1956 content has been released into the circulation via exosomes.

Altogether, these findings provide a novel mechanism in which cardio-renal exosome-derived miRNA-1956 regulates the activation of paracrine VEGF signaling in ADMSCs after MI. Moreover, we highlight the important role of exosomes in intercellular communication between different tissues/organs, and in the regulation of endogenous stem cells in pathological conditions. Therefore, targeting exosome-based miRNA transfer between organs/tissues may represent a novel therapeutic strategy for tissue regeneration and repair after myocardial ischemia.

## Supplementary Material

Supplementary figures and tables.

## Abbreviations

ADMSCs: adipose derived mesenchymal stem cells
GO: gene ontology
KEGG: kyoto encyclopedia of genes and genomes
miRNAs: microRNAs
MI: myocardial infarction
Con Exo: control exosomes
MI Exo: myocardial infarction exosomes
