# Supplementary material for: Cardio-renal Exosomes in Myocardial Infarction Serum Regulate Proangiogenic Paracrine Signaling in Adipose Mesenchymal Stem Cells
Source: Theranostics. 2020 Jan 1;10(3):1060–73. doi: 10.7150/thno.37678 (PMC6956822; doi:10.7150/thno.37678)
Supplement: Supplementary file 1 — Supplementary figures and tables. [file thnov10p1060s1.pdf]

Supplementary Information for

Cardio-renal Exosomes in Myocardial Infarction Serum Regulate Proangiogenic Paracrine Signaling in Adipose Mesenchymal Stem Cells

Lei Gao[1,2]*, Shuya Mei[3]*, Shuning Zhang[4]*, Qing Qin[4]*, Hao Li[5], Yiteng Liao[5], Huimin Fan[1,2]✉, Zhongmin Liu[1,2]✉, Hongming Zhu[1,2]✉

1. Research Institute of Heart Failure, Shanghai East Hospital, Tongji University School of Medicine, Shanghai, China
2. Translational Medical Center for Stem Cell Therapy & Institute for Regenerative Medicine, Shanghai East Hospital, Tongji University School of Medicine, Shanghai, China
3. Department of ICU, South Campus, Renji Hospital, School of Medicine, Shanghai Jiao Tong University, Shanghai, China
4. Department of Cardiology, Zhongshan Hospital, Fudan University, Shanghai Institute of Cardiovascular Disease, Shanghai, China.
5. Department of Cardiology, Shanghai Tenth People's Hospital, Tongji University School of Medicine, Shanghai, China

*These authors contributed equally to this work.

✉ Corresponding authors: Hongming Zhu: zhm@tongji.edu.cn, Huimin Fan: frankfan@tongji.edu.cn, and Zhongmin Liu: liu.zhongmin@tongji.edu.cn

Table S1 Sequences of miR-1956 mimic and inhibitor.

miRNA	Sense (5' – 3')	Antisense (5' – 3')
micrOFFTM inhibitor Negative Control	CAGUACUUUUGUGUAG UACAAA	/
micrONTM mimic Negative Control	UUUGUACUACACAAAA GUACUG	CAGUACUUUUGUGUAG UACAAA
micrONTM mmu-miR-1956 mimic	AGUCCAGGGCUGAGUC AGCGGA	UCCGCUGACUCAGCCCU GGACU
micrOFFTM mmu-miR-1956 inhibitor	UCCGCUGACUCAGCCC UGGACU	/

Table S2 Primers for real-time PCR analysis.

Gene	Forward	Reverse
Cd31	AGTTGCTGCCCATTCATCAC	CTGGTGCTCTATGCAAGCCT
Vegf	AATGCTTTCTCCGCTCTGAA	GCTTCCTACAGCACAGCAGA
Notch-1	GATGGCCTCAATGGGTACAAG	TCGTTGTTGTTGATGTCACAGT
Gapdh	AGGTCGGTGTGAACGGATTTG	GGGGTCGTTGATGGCAACA

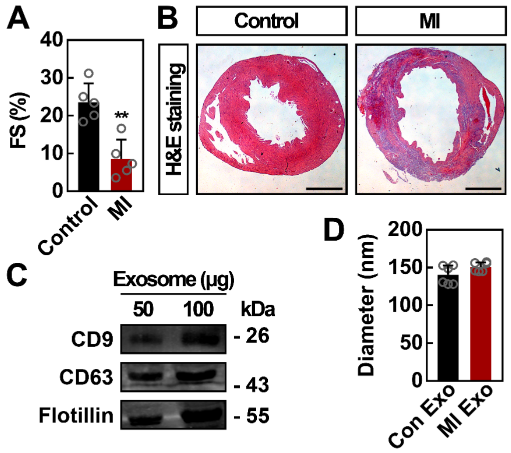

Figure S1. Cardiac function assessment after myocardial infarction. (**A**) Echocardiographic measurements of FS of control mice and mice with MI for 3 days. MI, myocardial infarction; FS, fractional Shortening. n = 5 mice per group. Data are shown as mean ± SD. *, significantly different from control; **, $p < 0.01$. (**B**) Representative images of H&E staining of control mice and mice with MI for 3 days. Scale bar, 1 mm. n = 3 mice per group. (**C**) Western blot analysis of exosomal marker proteins including CD9, CD63, and Flotillin. The total protein amount of exosomal lysate was 50 μg and 100 μg, respectively. Representative images of two independent experiments are shown. (**D**) Quantification of the average diameter of Con Exo and MI Exo isolated from an equal volume of serum. n = 6 mice per group. Data are shown as mean ± SD. Unpaired Student's t-test was performed (**A** and **D**).

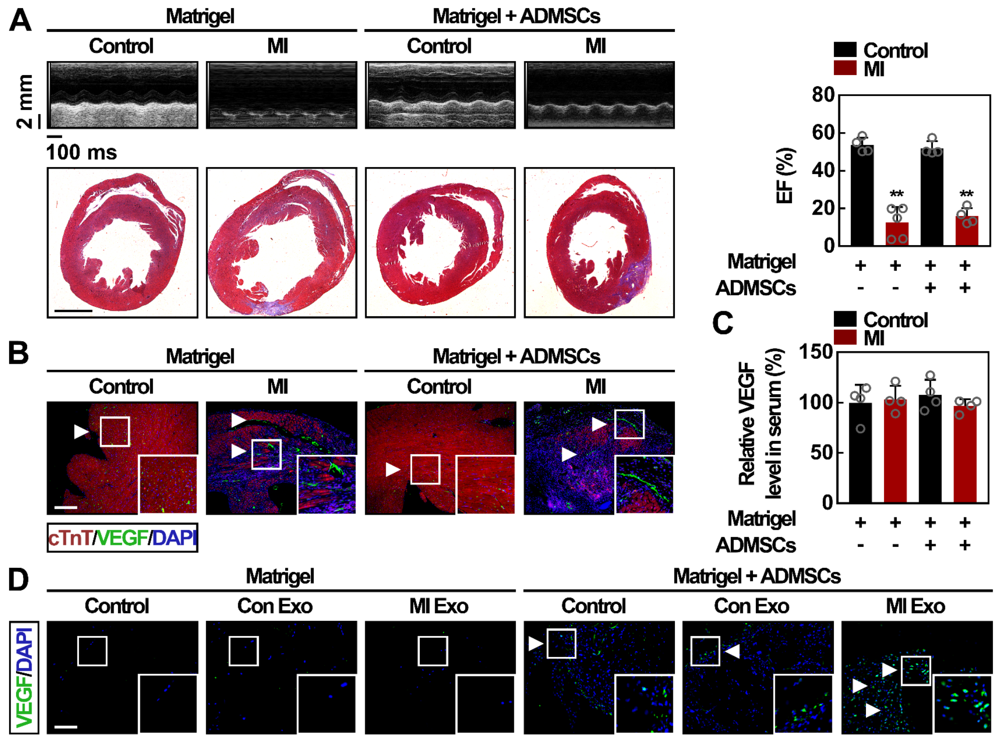

Figure S2. Circulating exosomes after MI and MI Exo promote the expression of VEGF in the Matrigel plugs. (**A**) Representative images of echocardiography and echocardiographic measurements of EF at Day 14. EF, ejection fraction. n = 4 mice per group at least. Data are shown as mean ± SD. *, significantly different from the control group bearing with cell-free Matrigel; **, $p < 0.01$. Representative images of Masson's trichrome-stained sections were exhibited. Scale bar, 1 mm. n = 4 mice per group. (**B**) Representative images of cTnT (red) and VEGF (green) expression in left ventricular free-wall sections. Nuclei were labeled with DAPI (blue). Scale bar, 200 µm; arrows indicate VEGF positive area. n = 4 mice per group. (**C**) The level of VEGF in the serum was assessed by ELISA. n = 4 mice per group. Data are shown as mean ± SD. *, significantly different from the control group bearing with cell-free Matrigel. (**D**) Representative images of VEGF (green) expression in Matrigel plug sections. Nuclei were labeled with DAPI (blue). Scale bar, 100 µm; arrows indicate VEGF positive area. n = 4 mice per group. One-way ANOVA followed by Tukey's post-test was performed (**A** and **C**).

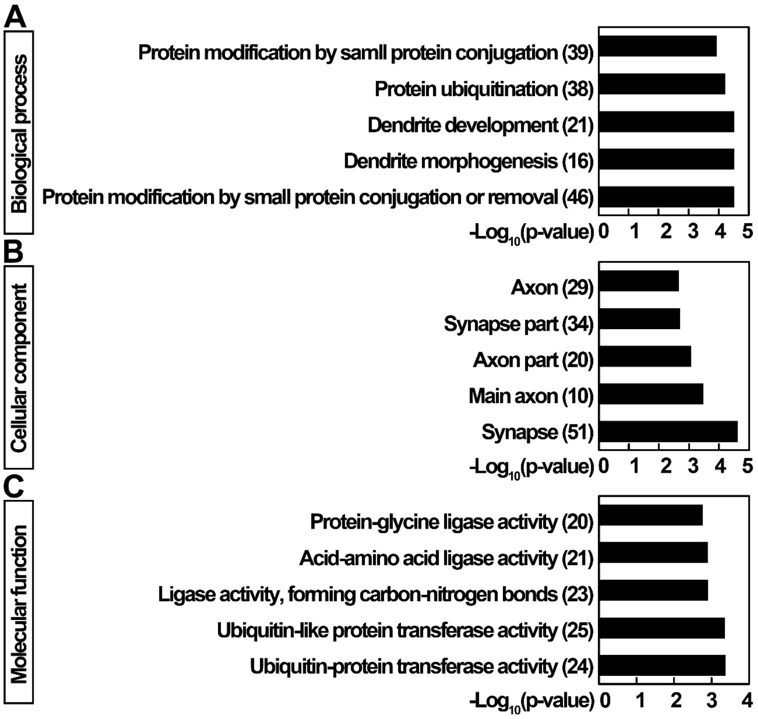

Figure S3. GO analysis of predicted target genes for the 8 significantly upregulated miRNAs in MI Exo. Top 5 enriched GO categories under molecular function (**A**), cellular component (**B**), and biological process (**C**) were shown.

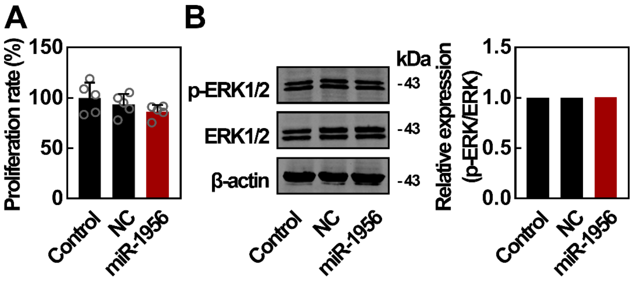

Figure S4. Overexpression of miR-1956 does not affect the cell viability of ADMSCs. After transfection with 50 nmol/L miR-1956 mimic for 3 days, the proliferation rate of ADMSCs was measured by CCK-8 assay (**A**), and the expression of intracellular p-ERK1/2 and ERK1/2 was determined by western blotting (**B**). Data are shown as mean ± SD. One-way ANOVA followed by Tukey's post-test was performed (**A**).
